# Effect of Inhomogeneous Broadening in Ultraviolet III-Nitride Light-Emitting Diodes

**DOI:** 10.3390/ma14247890

**Published:** 2021-12-20

**Authors:** Friedhard Römer, Martin Guttmann, Tim Wernicke, Michael Kneissl, Bernd Witzigmann

**Affiliations:** 1 Lehrstuhl für Optoelektronik, Friedrich-Alexander-Universität Erlangen-Nürnberg, Cauerstraße 7, D-91058 Erlangen, Germany; bernd.witzigmann@fau.de; 2 Institut für Festkörperphysik, Technische Universität Berlin, Hardenbergstraße 36, D-10623 Berlin, Germany; martin.guttmann@physik.tu-berlin.de (M.G.); tim.wernicke@physik.tu-berlin.de (T.W.); kneissl@physik.tu-berlin.de (M.K.)

**Keywords:** light emitting diode, III-nitride, inhomogeneous broadening, efficiency, numerical modelling

## Abstract

In the past years, light-emitting diodes (LED) made of GaN and its related ternary compounds with indium and aluminium have become an enabling technology in all areas of lighting. Visible LEDs have yet matured, but research on deep ultraviolet (UV) LEDs is still in progress. The polarisation in the anisotropic wurtzite lattice and the low free hole density in p-doped III-nitride compounds with high aluminium content make the design for high efficiency a critical step. The growth kinetics of the rather thin active quantum wells in III-nitride LEDs makes them prone to inhomogeneous broadening (IHB). Physical modelling of the active region of III-nitride LEDs supports the optimisation by revealing the opaque active region physics. In this work, we analyse the impact of the IHB on the luminescence and carrier transport III-nitride LEDs with multi-quantum well (MQW) active regions by numerical simulations comparing them to experimental results. The IHB is modelled with a statistical model that enables efficient and deterministic simulations. We analyse how the lumped electronic characteristics including the quantum efficiency and the diode ideality factor are related to the IHB and discuss how they can be used in the optimisation process.

## 1. Introduction

Lighting technology has been steadily changing towards solid state lighting since about ten years ago. This evolution has been enabled by visible light-emitting diodes (LED) made of Gallium Nitride (GaN) and its related compounds Indium Gallium Nitride (InGaN) and Aluminium Gallium Nitride (AlGaN) which cover a band gap from 0.69 eV $\leq E_{g}\leq$ 6.1 eV [1]. Recently, Blue GaN/InGaN LEDs have had an internal quantum efficiency (IQE) in excess of 90% [2]. Besides lighting, there is an increasing interest in III-nitride LEDs for sensing [3,4]. The emerging deep ultraviolet (UV) LEDs are in high demand for water purification, medical treatment, and material processing [5]. However, deep UV LEDs are still subject of research because their efficiency does not yet exceed 10% [6].

The realisation and optimisation of LEDs for a specific application is often complicated by the opaque physical processes in the active region. Lumped characteristics such as the current versus voltage (I/V) curve and the emission spectrum are usually easily accessible but do not permit immediate conclusions on the active region physics. Experiments on the microscopic scale such as secondary ion mass spectroscopy are often expensive as well as destructive.

Physical modelling of the III-nitride LEDs helps understand the opaque active region physics. Here, we propose predictive physical modelling to elucidate the physical processes in the active region through the lumped characteristics in order to enable a detailed optimisation. This aim requires a tight interaction between experiment and physical modelling to calibrate the model parameters.

III-nitride LEDs designed for high efficiency feature a multi-quantum well (MQW) active region to mitigate the efficiency droop [7] related to the Auger recombination and electron leakage [8,9,10]. Because of the polarisation in the wurtzite crystal lattice and in absence of lattice matching quantum wells (QW) in III-nitride, LEDs are usually thin extending only over few atomic layers. This makes their properties more prone to compound disorder originating from the hetero interfaces. Recent findings suggest that for UV laser operation thick III-nitride QWs may be advantageous [11].

**Figure 1:**
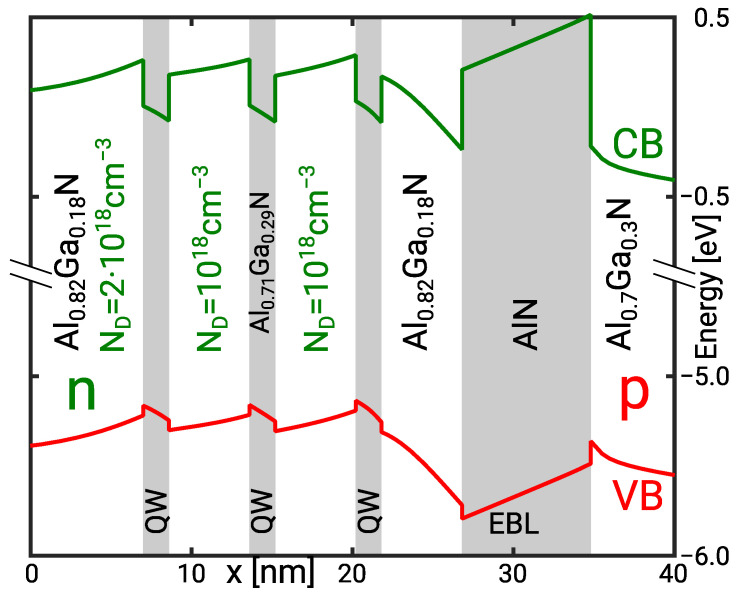
Conduction and valence band edges in the triple QW active region of a deep UV LED under forward bias. The potential gradient in the barriers and QWs is due to piezoelectric and spontaneous polarisation. The curvature of the band edge in the barriers is related to free carrier screening and thus the barrier doping.

The threefold MQW active region of a simulated deep UV LED is illustrated in Figure 1. Increasing the number of quantum wells (QW) increases the active volume and reduces the contribution of the Auger recombination by means of the lower average electron and hole density. However, the MQW is only effective if the radiative recombination rate is equally distributed amongst the QWs. This sets an upper limit for the number of QWs in an MQW active region.

The carrier transport in the III-nitride material system is subject to the spontaneous and piezoelectric polarisation [12] and the high acceptor ionisation energy [13]. The resulting polarisation fields and the low hole injection efficiency make the transport in the MQW inherently asymmetric. The design of the MQW region including the doping profiles is therefore highly critical to achieve a balanced quantum well luminescence. The lower hole injection efficiency requires a good electron confinement in the active region. The confinement is usually realised by means of an electron blocking layer (EBL).

The operation of LEDs is affected by statistical fluctuations of the ternary compound. The inhomogeneous broadening (IHB) of the emission spectrum presents an indirect experimental evidence of this compound disorder. The fluctuation could be visualised in QWs [14,15] recently. Theoretical studies reveal a rather large impact of compound fluctuation on the carrier transport and the luminescence in an MQW. Atomistic simulations show a strong localisation of carriers [15,16] leading to enhanced Coulomb interaction. Tight binding calculations demonstrate a reduced band gap [17] and momentum matrix element [18] in polar III-nitride quantum wells affecting the radiative recombination efficiency. A recent theoretical study states that the compound fluctuation [19] enhances hole transmission in an MQW. All these studies suggest that apart from the IHB the entire LED operation is affected.

The impact of compound disorder on the III-nitride LED operation has been investigated recently by M. Filoche, M. Piccardo, and C. K. Li, et al. [20,21,22]. The proposed model is based on percolation transport in a potential landscape formed by the compound disorder. The potential landscape may be generated by a random process but also from experimental data. This approach enables the arguably most accurate physical description of transport in presence of compound disorder. In the context of the physical model based analysis the potential landscape approach is less practical because it is either not deterministic because of the random landscape or requires expensive experimental data. In addition, the potential landscape model is restricted to a numerically expensive three dimensional simulation. It does not perform well in optimisation or calibration.

In this work, we investigate the effect of compound disorder with a statistical model instead. A statistical modelling of the compound disorder seen through IHB has been considered in the microscopic modelling of the luminescence [23]. We have embedded statistical compound disorder modelling into a multi-scale multi-population carrier transport simulator to study its influence on the electronic operation. Details are given in Section 4.1 and Section 4.2.

Using the transport simulator with the statistical disorder model, we analyse the effect of the IHB on the IQE, the I/V curve, and the emission spectrum. In Section 2.1, we demonstrate that the IHB not only affects the spectral emission, but has also an immediate influence on the I/V curve and the ideality factor. The detailed study is enabled by an LED circuit model [24]. Details are given in Section 4.3. Simulation results for an MQW series are presented in Section 2.2 and compared to experiments. In Section 3, we assess the significance of the IHB for the LED operation.

## 2. Results

In this section, we analyse the effect of the composition disorder on the lumped characteristics demonstrating that the I/V curve facilitates conclusions on the physical processes in the active region. First, we investigate a single quantum well (SQW) LED in absence of non-radiative recombination and incomplete dopant ionisation as a simple model case. The findings are applied when comparing experimental data and physical modelling of a III-nitride LED with three QWs emitting in the deep UV.

Attention is paid to the I/V curve, IQE, and emission spectrum in the analysis. The I/V curve is inexpensive, closely related to the active region transport, and less subject to systematic errors than the IQE curve. The emission spectrum on the other hand side is hardly related to the carrier transport but a clear sign of spectral broadening.

The interpretation of the I/V curve is impeded by the contact voltage drop, the scaling of the current with the device area, and its relative insensitivity. Therefore, the discussion focuses on the ideality factor and the normalised resistances in the equivalent circuit as outlined in Section 4.3.

### 2.1. Single Quantum Well

The investigated SQW device has a 2.2 nm wide polar AlGaN QW confined by 10 nm wide intrinsic AlGaN barriers on an AlN substrate. An electron blocking layer is not included to keep the structure simple. The doping density in the quasi neutral n- and p-regions is $N_{D}=N_{A}=10^{18}$ cm${}^{-3}$. As incomplete ionisation has been disabled these values correspond to the free electron and hole density, respectively. Shockley–Read–Hall and Auger recombination are disabled to remove their influence on the QW recombination. Leakage due to minority recombination at the contacts is the only loss channel. Two different configurations are investigated: an Al${}_{0.71}$Ga${}_{0.29}$N/Al${}_{0.82}$Ga${}_{0.18}$N UVC QW and an Al${}_{0.47}$Ga${}_{0.53}$N/Al${}_{0.61}$Ga${}_{0.39}$N UVB QW. Without IHB, the UVC QW shows predominant TM-polarised emission [25].

The broadening of the conduction and valence sub-band energies is subject to their shift with the composition parameter *x*. The IHB of the conduction band is subject to the broadening energy $\sigma_{c}$. The broadening energy values $\sigma_{\mathrm{lh}}=\sigma_{\mathrm{hh}}$ hold for the light and heavy hole bands, respectively. The large negative crystal field splitting energy in AlN leads to a significantly weaker shift of the split off valence band with the compound parameter *x* [26]. Thus, the split off band broadening energy $\sigma_{\mathrm{so}}$ is smaller than $\sigma_{\mathrm{lh}}$ or $\sigma_{\mathrm{hh}}$. Considering the shift of the valence and conduction band offsets in the UVC QW the ratio of the electron broadening energy to light/heavy hole and split-off broadening energy is $\sigma_{c}\approx 2\sigma_{\mathrm{hh}}\approx 2\sigma_{\mathrm{lh}}\approx 4\sigma_{\mathrm{so}}$. Introducing the broadening energy $\sigma =\sigma_{c}+\sigma_{\mathrm{hh}}$ the sub-band broadening energies become

(1) $$\sigma_{e}=2/3\sigma ,\;\sigma_{\mathrm{lh}}=\sigma_{\mathrm{hh}}=1/3\sigma ,\;\mathrm{and}\;\sigma_{\mathrm{so}}=1/6\sigma$$

It has been pointed out that the broadening depends on the spatial averaging of the fluctuations related to the localisation length of the carriers [14,16,22,23]. As the localisation length may be different for each sub-band, there is arguably an effect on the ratio in Equation (1). We do not consider the localisation effect explicitly in absence of information on the length scale of the fluctuations and the uncertainty of the localisation length. To the end, the broadening energy values are fit parameters that are calibrated with experimental data.

**Figure 2:**
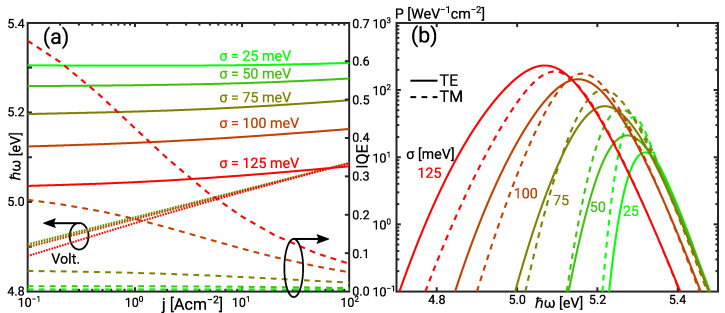
(**a**) Voltage, IQE, and expectation value of the photon energy versus the current for the UVC QW as function of the IHB energy. (**b**) TE and TM emission spectrum as function of the IHB energy.

Figure 2 shows the simulation results for the simplified UVC SQW LED. The mean photon energy decreases with increasing IHB. The shift increases quadratically with the broadening energy for parabolic bands in the Boltzmann limit, $\Delta \hbar \omega =-\sigma^{2}/\left(2k_{b}T\right)$. The increase of the mean photon energy with increasing current is due to the phase space filling of the low energy tail states. TM-polarised emission dominates as expected for an IHB as low as σ=25 meV. TE polarisation dominates with increasing IHB. The change in the polarisation results from the reduced broadening of the split-off bands. The TE emission spectra show a low energy tail despite the symmetric Gaussian broadening. It is noted that this tail is related to the asymmetry of the Fermi distribution and does not include all effects contributing to the Urbach tail. The low-energy tail is therefore more pronounced in the experiment.

**Figure 3:**
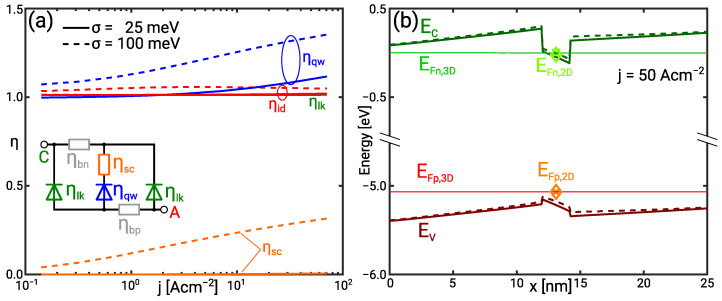
(**a**) Normalised scattering ($\eta_{\mathrm{sc}}$), quantum well ($\eta_{\mathrm{qw}}$), and leakage ($\eta_{\mathrm{lk}}$) resistance as well as the ideality ($\eta_{\mathrm{id}}$) in the UVC SQW as a function of the current for the IHB energy σ=25 meV (solid) and σ=100 meV (dashed). (**b**) Conduction and valence band as well as 3D and 2D QFLs in the UVC SQW for the IHB energy σ=25 meV (solid/cross) and σ=100 meV (dashed/diamond) at j=50 A cm${}^{-2}$.

The increase of the mean photon energy with decreasing IHB is attended by a decrease of the IQE in Figure 2a. This can be understood considering the equivalent circuit of the UVC SQW LED shown in Figure 3a and further explained in Section 4.3. Minority recombination and the radiative recombination make up the bipolar current. Figure 3b shows that the quasi Fermi level (QFL) distance, and thus bias voltage is nearly independent of the IHB. At constant QFL distance, the radiative recombination rate decreases exponentially with increasing photon energy in the Boltzmann limit. As minority recombination does not depend on the photon energy, it becomes the dominant contribution with increasing mean photon energy.

The redistribution of the current leads to the decrease of the IQE. It is caused by the increase of the normalised quantum well and scattering resistance while the normalised leakage resistance remains constant $\eta_{\mathrm{lk}}\approx 1$. This is illustrated in Figure 3a. The increase of the quantum well resistance is due the phase space filling. Transitions below the nominal sub-band transition energy enhance the effect of the phase space filling explaining the increase of $\eta_{\mathrm{qw}}$ with the IHB [27]. However, the increased quantum well resistance is not reflected in the diode ideality factor because radiative recombination still presents the minor current contribution. Thus, the I/V curves also show only a weak dependence on the IHB.

The scattering resistance $\eta_{\mathrm{sc}}$ accounts for the interaction of continuum and bound carriers in the QW [28]. Bound electrons and holes are nearly in thermal equilibrium with the continuum carriers in the rather shallow UVC QW. Figure 3b demonstrates that the respective 2D and 3D QFLs virtually coincide. The scattering resistance vanishes for σ=25 meV but takes values $\eta_{\mathrm{sc}}>0$ for σ=100 meV indicating a separation of continuum and bound QFLs. This separation can be understood looking at the band occupation in the presence of IHB. It is noted that the effect of the IHB may be regarded as a shift of band edge or, alternatively, as an increase of the density of states (DOS). Either the Fermi level separation increases at constant density, or the density increases at constant Fermi level with the increase of the DOS. The actual solution is a balance of both effects because the shift of the band edges between σ=25 meV and σ=100 meV in Figure 3b demonstrates an increase of the QW electron density. The hole density increases not as much as the electron density. The net increase of the negative charge causes the band edge shift.

**Figure 4:**
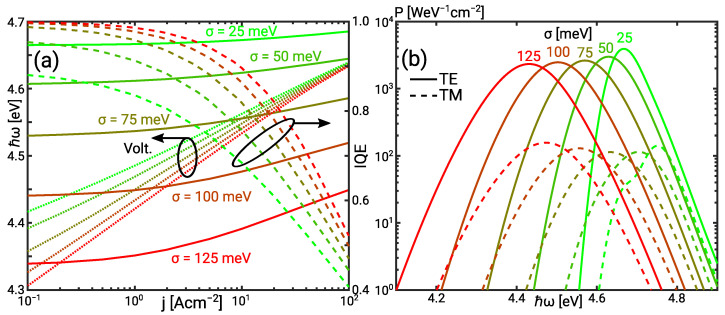
(**a**) Voltage, IQE, and expectation value of the photon energy versus the current for the UVB QW as function of the IHB energy. (**b**) TE and TM emission spectrum as function of the IHB energy.

**Figure 5:**
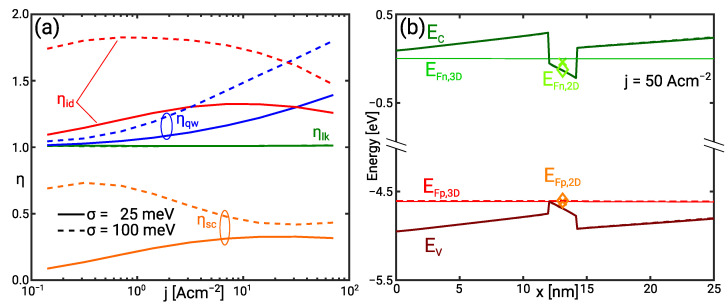
(**a**) Normalised scattering ($\eta_{\mathrm{sc}}$), quantum well ($\eta_{\mathrm{qw}}$), and leakage ($\eta_{\mathrm{lk}}$) resistance as well as the ideality ($\eta_{\mathrm{id}}$) in the UVB SQW as a function of the current for the IHB energy σ=25 meV (solid) and σ=100 meV (dashed). (**b**) Conduction and valence band as well as 3D and 2D QFLs in the UVB SQW for the IHB energy σ=25 meV (solid/cross) and σ=100 meV (dashed/diamond) at j=50 A cm${}^{-2}$.

Figure 4a illustrates that the IQE approaches 1 in the low current regime if the photon energy is in the range of the bias voltage. This confirms the dependence of the QW current on the photon energy. Leakage is not the dominant current contribution so that the normalised QW and scattering resistance both determine the ideality factor depicted in Figure 5a. The normalised QW resistance $\eta_{\mathrm{qw}}$ is generally higher than in the UVC SQW which is related to the higher radiative recombination current rather than a stronger effect of the IHB.

The normalised scattering resistance $\eta_{\mathrm{sc}}$ is also larger than in the UVC QW. Figure 5b shows an increasing separation of continuum and bound electron QFL with increasing IHB. For σ=100 meV the electron Fermi level separation is as high as $E_{\mathrm{Fn}},3D-E_{\mathrm{Fn}},2D\approx 5k_{b}T$. The Fermi level separation eliminates the effect of the bound population on the scattering. This buffering capability of the scattering is discussed in [24] and is reflected in the decrease of $\eta_{\mathrm{sc}}$ while $\eta_{\mathrm{qw}}$ increases.

The significant increase of the ideality factor with the IHB energy is reflected in the I/V curve set in Figure 4a. The curves converge in the high current limit where the effect of the IHB vanishes with increasing leakage. This is also reflected in the decrease of the ideality factor in the high current limit. The enhancement of the blue shift of the photon energy with increasing current is seen through increased phase space filling by the IHB. Figure 4b shows insignificant TM-polarised emission for all IHB energies in the UVB LED despite the contribution of multiple transitions. Here, the mean energy of the TM-polarised emission is clearly higher than the mean energy of the TE-polarised emission. At σ=25 meV, the TM-polarised spectrum shows a low energy shoulder which coincides with the TE-spectrum. The shoulder is due to the minor TM emission from the heavy and light hole band [29] and is hidden with increasing IHB.

### 2.2. Multi-Quantum Well

The simulation of the simplified LED structure demonstrates a strong impact of the IHB on the LED characteristics. The reduction of the emission energy is attended by an decrease of the forward voltage and an increase of the IQE, especially for current densities below j<10 A cm${}^{-2}$. This effect can be observed for all QW mole fractions though the impact on the electronic operation depends much on the design of the active region.

Here, we investigate a realistic deep UV LED with an emission wavelength near 245 nm. We analyse the effect of the QW width on the electron leakage and thus the IQE with the transport model presented in Section 4. The active region of the sample structure is illustrated in Figure 1. A quasi-neutral region with a Si donor doping of $N_{D}=4\times 10^{18}$ cm${}^{-3}$ is followed by a 40 nm wide n-side barrier. The nominal quantum well and barrier compounds are Al${}_{0.71}$Ga${}_{0.29}$N and Al${}_{0.82}$Ga${}_{0.18}$N, respectively. The band gap bowing energy is b=1.1 eV [30]. The quantum well width is varied from $d_{\mathrm{QW}}=0.5$ nm to 2.8 nm corresponding with one to five unit cells. Holes are injected through a 25 nm wide Mg-doped Al${}_{0.7}$Ga${}_{0.3}$N layer next to the 8 nm wide AlN electron blocking layer (EBL). The quasi neutral region on the p-side consists of a 150 nm wide AlGaN superlattice followed by a 10 nm GaN contact layer with an Mg doping of $N_{A}=10^{19}$ cm${}^{-3}$. The superlattice has been replaced with an effective Al_0.37_Ga_0.63_N compound because it does not affect the active region.

Donors as well as acceptors are subject to incomplete ionisation and proximity correction [31]. The ionisation energy of Mg acceptors increases with the Al content exceeding $E_{A}>0.6$ eV for AlN [13]. Muhin et al. [32] report an acceptor ionisation energy $E_{A,0}=510\pm 20$ meV in an Al${}_{0.71}$Ga${}_{0.29}$N/Al${}_{0.65}$Ga${}_{0.35}$N short period superlattice. Therefore, the EBL as well as the p-side barrier do not contribute to the hole injection. Simulations show that the free hole density in the hole injection layer is as low as $p\approx 2\times 10^{16}$ cm${}^{-3}$. SRH recombination in the QWs is subject to a threading dislocation density of $N_{\mathrm{td}}=2\times 10^{9}$ cm${}^{-2}$ [33]. The calibrated coefficients for the Auger recombination, the scattering, the piezoelectric polarisation, and spontaneous polarisation of III-nitride LEDs emitting in the blue spectral range [10] are used in absence of data for deep UV LEDs.

**Figure 6:**
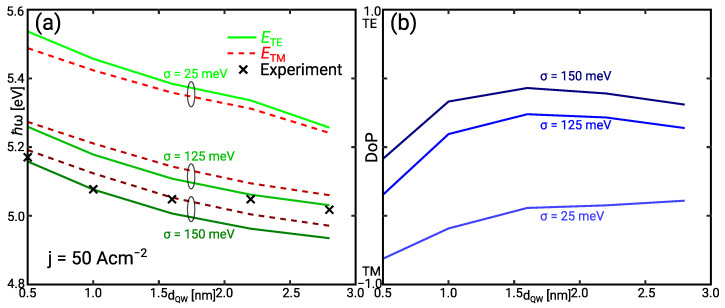
(**a**) Simulated mean photon energy of the TE (solid) and TM (dashed) emission. The crosses show the mean photon energy calculated from the measured spectra. (**b**) Simulated degree of polarisation DoP$=\left(P_{\mathrm{TE}}-P_{\mathrm{TM}}\right)/\left(P_{\mathrm{TE}}+P_{\mathrm{TM}}\right)$. All data are shown for the current density j=50 A cm${}^{-2}$ versus the quantum well width $d_{\mathrm{QW}}$ varying the IHB energy σ. The experimentally observed mean photon energy can be explained with an IHB energy between 125 meV ≤σ≤ 150 meV.

Figure 6a shows the simulated mean TE and TM photon energy together with mean photon energy of the measured spectra. These values can be explained assuming an IHB energy between 125 meV ≤σ≤ 150 meV. The TM polarised emission vanishes in the spectral characterisation due to the lower extraction efficiency. Only the mean TE photon energy is considered for the comparison. The decrease of the mean TE photon energy with the IHB is stronger than the decrease of the TM photon energy because of the different IHB energy values for the light/heavy hole band and the split-off band. The simulated degree of polarisation (DoP) in Figure 6b shows dominant TE polarisation for $d_{\mathrm{QW}}\geq 1$ nm corresponding with experimental findings [34].

**Figure 7:**
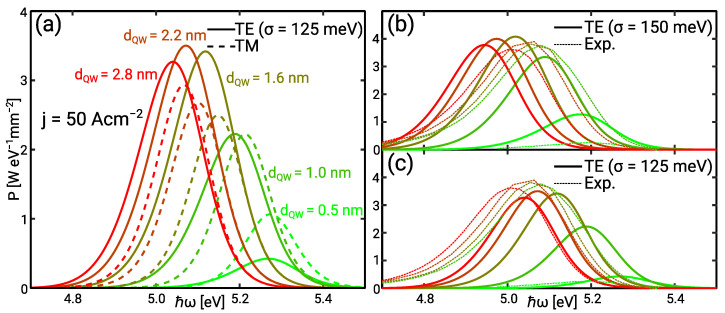
(**a**) Simulated TE (solid) and TM (dashed) emission spectra for the IHB energy σ=125 meV. (**b**) Simulated TE emission spectra for σ=125 meV (solid) and measured emission spectra (dashed). (**c**) Simulated TE emission spectra for σ=150 meV (solid) and measured emission spectra (dashed). All data are shown for the current density j=50 A cm${}^{-2}$ varying the quantum well width $d_{\mathrm{QW}}$. The measured spectra are scaled.

Simulated TE and TM emission spectra are shown in Figure 7a. The contribution of the TE emission increases due to the reduced broadening of the split-off band with increasing IHB energy. The simulated TE spectra and the measured spectra illustrated in Figure 7b for σ=125 meV and Figure 7c for σ=150 meV show the same trend of the red shift and relative output power with increasing QW width. The intensity of the measured spectra has been scaled with the same factor keeping the relations in the QW width variation series. The weak measured TE emission spectrum for $d_{\mathrm{QW}}=0.5$ nm is also observed in the simulation. The TM emission is less affected but does not contribute significantly to the measured spectrum as discussed above. The width and high energy slope of the simulated spectra match the experiment quite well. The low energy tail of the simulated spectra is solely due to the IHB. Other effects contributing to the Urbach tail are neglected. Thus, it is less pronounced than in the experiment.

If the IHB is neglected, the mean photon energy can only be matched assuming that the QW Al mole fraction is much lower than anticipated. However, simulations show that if the QW Al mole fraction is reduced the red shift with the QW width does not match the experiment any more. In addition, the spectral function does not match without IHB. Therefore, the DOS broadening seen though the IHB presents a stringent explanation for the experimentally observed spectral emission.

**Figure 8:**
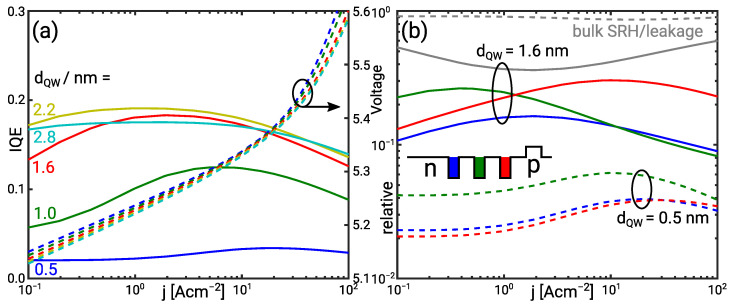
(**a**) IQE (solid) and I/V (dashed) curves versus the QW width for σ=125 meV (**b**) Relative contribution of each QW and minority/bulk SRH recombination to the total current for the 0.5 nm (dashed) and 1.6 nm (solid) wide QW. The inset illustrates the position of the QW.

The operation of the active region has been analysed for the IHB energy value σ=125 meV. The simulated IQE for different quantum well widths are shown in Figure 8a. The 1.6 nm and 2.2 nm wide quantum wells yield the highest IQE $\eta_{\mathrm{int}}\approx 0.2$. The 0.5 nm wide quantum well shows a decay in the IQE which conforms well with experimental observations. Note that the decay is less pronounced than in the experiment because the TM emission contributes to the simulated IQE. Leakage is made up of bulk SRH and and contact minority recombination. It presents the dominant current contribution as illustrated in Figure 8b.

**Figure 9:**
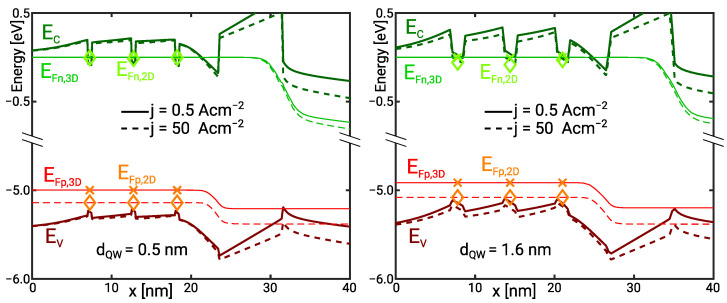
Conduction and valence band and 2D/3D QFLs in the active region of the MQW LED for the 0.5 nm QW and the 1.6 nm QW. The bands and QFLs are shown for j=0.5 A cm${}^{-2}$ (solid/cross) and j=50 A cm${}^{-2}$ (dashed/diamond).

The high leakage suggests a low electron or hole injection efficiency into the QW. This is investigated by means of the band diagrams in forward bias which are shown in Figure 9. As illustrated by the Fermi levels bound and continuum holes are always nearly in thermal equilibrium. Electrons are in thermal equilibrium at j=0.5 A cm${}^{-2}$ in the active region with 1.6 nm wide QWs. However, they are not in thermal equilibrium at a current density of j=50 A cm${}^{-2}$. Enhanced hole injection increases the hole density while the electron density in the n-side and mid QW remains nearly constant because of the barrier doping. Therefore, the bound electron QFL shows a negative shift seen though the non-equilibrium between bound and continuum electrons. The electron accumulation in the p-side barrier impedes the QFL shift in the p-side QW. The accumulation of electrons in the p-side QW and the hole injection lead to the re-distribution of the luminescence from the n-side and mid QW to the p-side QW with increasing current. This effect is illustrated in Figure 8b. The re-distribution enables the balanced luminescence distribution amongst the QWs [24].

Electrons are nearly in thermal equilibrium over the whole current range in the active region with the 0.5 nm wide QWs. The carrier injection into the QWs is limited by high nominal sub-band energies and a vanishing Stark effect. Figure 8b shows that the relative contribution of each QW to the total current remains nearly the same over the whole current range. The low electron injection efficiency is seen through nearly 100% electron leakage. The radiative recombination in the p-side QW is reduced because here the electron wave function extends into the p-side barrier reducing the optical matrix element. A redistribution as in the active region with 1.6 nm wide QWs cannot be observed.

## 3. Discussion

The composition disorder in quantum wells (QW) is considered as the cause of the inhomogeneous broadening (IHB) of the spectral emission [35]. Atomic-scale microscopy [14] experiments confirm the disorder. The carrier transport and luminescence in thin film structures subject to composition disorder has been investigated theoretically with a drift-diffusion approach enhanced by a localised landscape potential function [20,22,36]. However, for predictive simulations and model-based design, a statistical description is advantageous because it is deterministic and more efficient. In this work the statistical modelling of the IHB is enabled by a Gaussian broadening of the sub-band level energies of the radiative transitions [23]. It has been integrated into the microscopic luminescence model based on the k·p-Schrödinger solutions. It acts like a broadening of the density of states (DOS) on the carrier transport level. We have integrated the DOS broadening into our multi-scale multi-population carrier transport simulator for analysing the effect of the IHB.

The model accuracy is arguably restricted by its limitation to the sub-band energies discarding other effects of the IHB, the symmetric Gaussian broadening which does not account for the Urbach tail, and neglecting the lateral carrier localisation. However, considering the broadening energies attributed to the sub-band transitions as fit parameters it is not necessary to include all the details into the model. The direct and indirect influence of the composition disorder on the sub-band energy levels dominates all other effects [23] and the model is described by three broadening parameters. To the end, the model demonstrates good agreement with experimental results.

We have investigated the interaction of the IHB on the operation of a single QW solving carrier transport and microscopic luminescence self-consistently. Different broadening energy values for light/heavy hole, and split-off bands in addition to the conduction band broadening energy have been used to account for the negative crystal field splitting in AlN. The mean photon energy reduces with increasing IHB due to the increasing contribution of states below the nominal sub-band transition energy. The decrease of the mean photon energy is seen though an increase of the IQE if the bimolecular recombination competes with minority recombination.

The IHB affects the I/V characteristics on many levels. An immediate effect is the enhanced phase space filling in the presence of the IHB which increases the ideality factor of the bimolecular recombination. The self-consistent modelling confirms the study based on the effective mass model [27]. The IHB has got another indirect effect on the I/V curve because it affects scattering into the QW through a realignment of the QW QFLs. The effect of the IHB on the scattering is seen though an increase of the ideality factor. It depends on the band offsets and thus the design in contrast to the phase space filling. In summary, the IHB increases the gradient of the I/V curve as illustrated in Figure 4. The reduction of the bias voltage in the low current limit is caused by the realignment of the QFLs. In real devices, the gross effect of the IHB on the I/V curve depends on a high IQE and may be mitigated in the presence of minority recombination or by competing recombination mechanisms. Deep UV LEDs suffer from a high series resistance which dominates the I/V curve in the high current regime and hides the effect of the IHB preventing the experimental detection.

III-nitride LEDs in the visible spectral range often demonstrate a turn on voltage lower than the anticipated photon energy which can be explained by the IHB. An enhanced ideality factor is not a clear evidence for IHB, though. There are competing mechanisms such as the SRH recombination [37], the luminescence re-distribution inside the MQW [24], and the most prominent series resistance.

The model has been verified comparing the simulated mean photon energy, degree of polarisation, and emission spectra to experimental results for a UVC MQW LED series with varying QW width. The measured mean photon energies of the QW series are lower than the values predicted by the simulation without IHB. They can be matched including IHB showing the same red shift with increasing QW width. The IHB model reproduces the shape of the measured spectra. The simulated low energy tail is less pronounced because the Gaussian broadening does not account for all effects contributing to the Urbach tail. However, the contribution of the Urbach tail to the cumulative radiative recombination is not considered to affect the electronic model.

Dominant TE Polarisation results if the broadening energy value of the split-off band is half as high as the broadening of the light/heavy hole band. The choice of the ratio is justified by the gradient of the crystal field splitting energy in the AlGaN compound [26]. To the end, the reduced broadening of the split-off band explains the enhanced TE-emission observed in the experiment. Though the estimation of the absolute IQE is impeded by the unknown external efficiency the simulation model correctly predicts the trend in the TE spectral power density.

The effect of the IHB on the emission spectrum has been noted early, but its effect on the operation of active optoelectronic devices has been studied only recently. Simulations show that the IHB is not immediately critical for the LED efficiency. If such a correlation has been observed it might be due point defects and threading dislocations involved with the compound disorder. SRH recombination due to threading dislocations presents a loss channel in III-nitride LEDs [38]. Regarding the interaction with minority recombination the IHB may even enhance the efficiency though at lower photon energy. The competition of the IHB with SRH and Auger recombination cannot be predicted and depends on the relevance of phase space filling for the latter loss mechanisms. The increased width of the emission spectrum might be advantageous for LED applications but reduces the maximum gain in Laser applications. However, the practical use is limited and subject to technological control.

The IHB model used in this work is limited to the radiative recombination and Fermi levels in the QWs. The compound disorder in the bulk material may have also an effect of the carrier transport due to the modification of the DOS. It is pointed out that the carrier transport across AlGaN barriers is not yet fully understood [39]. The compound disorder might contribute to the explanation of the experimental results.

## 4. Materials and Methods

This section outlines the methods and models for the physical simulation of III-nitride LEDs. In the context of analysing the characteristic curves the physical modelling of the light generation in III-nitride LEDs is restricted to the electron and hole transport as well as the light matter interaction. The optical wave propagation model is not considered explicitly here because its influence in thin film LEDs restricts to a linear scaling factor relating the external efficiency to the IQE. Photon recycling as well as spontaneous emission enhancement are considered to have a minor impact.

Drift-diffusion transport models are in wide use for the design and analysis of III-nitride LEDs. There exist an number of commercial products [33,40] as well as academic simulators [22,41,42] which have been applied in this context.

### 4.1. Carrier Transport Model

**Figure 10:**
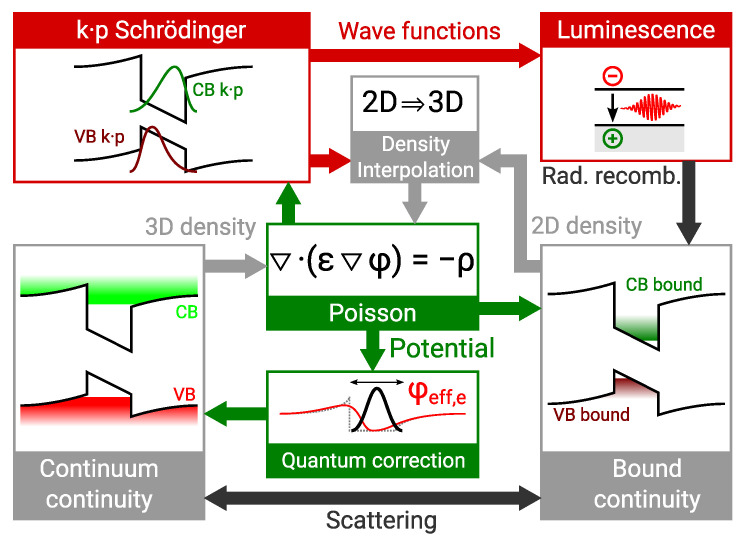
Schematic illustration of the multi-scale drift-diffusion simulator operation.

The carrier transport simulator used in this work is based on the drift-diffusion approach. It has been enhanced to account for non-equilibrium distribution and quantisation in QWs, microscopic luminescence, and the carrier transport in the presence of piezoelectric polarisation. A schematic of the simulation is shown in Figure 10. Carriers are separated in multiple populations which are subject to distinct continuity equations. There exist one continuum electron and hole population and as many bound electron and hole populations as there are QWs in the device. Continuum carriers are subject to the drift-diffusion equations in all spatial dimensions. Bound carriers are subject to drift-diffusion only in the transversal directions of the QW plane. Each population is subject to a distinct quasi Fermi level so that thermal equilibrium is not enforced between the populations. Instead, bound and continuum populations are coupled by a dynamic scattering model [43]. Non-equilibrium Green’s function simulations show that the multi population approach presents a good approximation for LEDs [44,45].

The bound carrier distribution in the normal direction of each QW is subject to a 6×6 k·p-Schrödinger problem [46] in the valence band and an effective mass Schrödinger problem in the conduction band. The bound and continuum continuity equations are solved together with the Poisson equation in a Newton iteration. Self consistency with the k·p-Schrödinger problem is achieved by means of a Gummel iteration. The transport problem reduces to one spatial dimension in thin film LEDs. The continuity equation in the QWs reduces to a recombination and capture balance equation.

Carrier transport is subject to the piezoelectric and spontaneous polarisation in the III-nitride material system. The piezoelectric polarisation used in the simulation is reduced to 50% of the theoretical values [12] to reproduce the experimental Stark shift of the mean photon energy [47].

The drift-diffusion model underestimates the barrier current in absence of the tunnelling contribution. This deficiency gives rise to the quantum corrected drift (QCD) model. Particles are assumed to be Gaussian wave packets of finite size subject to an average potential [33,48]. Only the continuum populations are subject to QCD because there are no potential barriers in the transversal direction of a QW. Colour-coded MQW experiments can be reproduced with QCD [28].

Shockley–Read–Hall (SRH) recombination, Auger recombination, and radiative (bimolecular) recombination contribute to the QW current. Threading dislocations give rise to the SRH recombination [38]. The QW Auger recombination model includes the envelope wave function overlap as proposed in [9]. The dynamic scattering model and the Auger recombination model depend on a phenomenological scattering time parameter and Auger coefficient, respectively, which were both calibrated with the characteristic curves of single QW LEDs emitting near 450 nm wavelength [10].

### 4.2. Inhomogeneous Broadening Model

Radiative recombination in the QWs is calculated with a microscopic model based on the k·p-Schrödinger solutions [29]. The microscopic luminescence model inherently accounts for phase space filling and the quantum confined Stark effect. Excitons are not included. The non-parabolic dispersion requires integration over the 2D momentum space $d^{2}k$ in the QW. The dispersion relation is isotropic for a polar (c-plane) QW so that the integration can be restricted to the radial direction. The dispersion relation enters the integration via the distribution function. Without IHB the spontaneous recombination rate for the polarisation $\vec{e}$ is defined by

(2) $$\begin{array}{c} r_{\mathrm{sp}}\left(\hbar \omega ,\vec{e}\right)=\frac{q^{2}\omega n}{\pi \epsilon_{0}m_{0}^{2}\hbar^{2}c^{3}}\\ \;\cdot \sum_{c,v}\int \frac{d^{2}k}{{\left(2\pi \right)}^{2}}\;L_{\mathrm{hb}}\left(E_{ck}-E_{vk}-\hbar \omega \right){\left|\vec{e}\cdot p_{\mathrm{cv}k}\right|}^{2}f\left(E_{ck}-E_{\mathrm{Fn}}\right)\left(1-f\left(E_{vk}-E_{\mathrm{Fp}}\right)\right) \end{array}$$

Here, $|\vec{e}\cdot p_{\mathrm{cv}k}{|}^{2}$ is the optical matrix element for the transition with the polarisation $\vec{e}$ between the conduction band *c* and the valence band *k*. The optical matrix element accounts for the k·p wave function overlap. $E_{ck}$ and $E_{vk}$ are the conduction and valence band dispersion relations, respectively, and *f* is the Fermi distribution. The sum runs over all conduction and valence band combinations. The $L_{\mathrm{hb}}$ stands for the homogeneous broadening function.

Modelling inhomogeneous broadening means ultimately the modelling of composition disorder and its effects on the band structure and carrier transport. We make several approximations for the statistical description. First, we limit the effect of the IHB to the QW composition and do not regard the effect on bulk material or the QW profile. The disorder is limited to a perturbation of the the zero order 6×6 k·p Hamiltonian operator [46] so that only the sub-band energies and not the dispersion relation or the optical matrix elements are affected. Thermal equilibrium is assumed in the transversal direction of the QW as proposed by Mounir et al. [23]. The carrier distribution in the QW is subject to a single QFL for electrons and holes.

Instead of calculating the perturbation of the sub-band level energy via the composition, the broadening of each sub-band is controlled by distinct broadening energy values for the conduction and valence band. In the AlGaN compounds different broadening energies apply for the light/heavy hole and split off bands because of the negative crystal field splitting energy in AlN [26]. We introduce the broadening energy $\sigma_{c}$ for the conduction band, $\sigma_{\mathrm{hh}}$, $\sigma_{\mathrm{lh}}$ for the heavy and light hole band, respectively, as well as $\sigma_{\mathrm{so}}$ for the split off band. Since the spin-orbit interaction energy and particularly its change with the composition is small compared to $k_{B}T$ we assume the same broadening energy values for the heavy and light hole bands.

The perturbation energy ε is considered to be subject to the distribution

(3) $$L_{\zeta }\left(\varepsilon \right)=\frac{1}{\sqrt{2\pi }\sigma_{\zeta }}\exp\left(-\frac{\varepsilon^{2}}{2\sigma_{\zeta }^{2}}\right)$$

Heavy/light hole and split-off bands are discriminated by the zero order perturbation Hamiltonian operator. The perturbation of the heavy/light and split off bands is expressed in terms of the valence band and crystal field splitting energy to calculate the broadening energy per band. With the operator

(4) $$H_{\mathrm{IHB}}^{\prime }=\mathrm{diag}\left(\sigma_{\mathrm{hh}},\sigma_{\mathrm{lh}},\sigma_{\mathrm{so}},\sigma_{\mathrm{hh}},\sigma_{\mathrm{lh}},\sigma_{\mathrm{so}}\right)$$

first order perturbation yields for valence band broadening energies

(5) $$\sigma_{v}=\langle \psi_{v0}|H_{\mathrm{IHB}}^{\prime }|\psi_{v0}\rangle$$

The broadening of the conduction sub-band energies is $\sigma_{c0}=\sigma_{c}$. It is noted that the perturbation of strain and deformation potentials is implicitly included.

**Figure 11:**
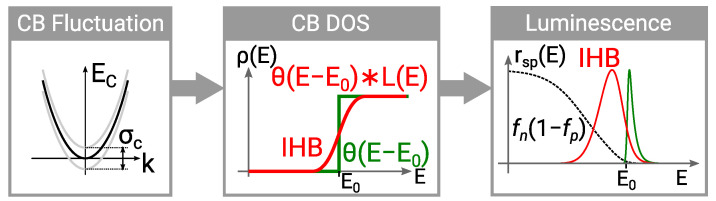
Schematic illustration of the luminescence and density of states broadening seen through compound disorder.

The sub-band energy perturbation affects the QFLs as well as the radiative recombination and can be regarded as a broadening of the density of states (DOS) as illustrated in Figure 11. The density integral is calculated in momentum space rather than the energy space. The dispersion relations enters the Fermi distribution. The integrating with respect to the perturbation energy yields the electron density

(6) $$n=2\sum_{c}\int \frac{d^{2}k}{{\left(2\pi \right)}^{2}}\int_{-\infty }^{\infty }d\varepsilon \;L_{c}\left(\varepsilon \right)f\left(E_{ck}+\varepsilon -E_{\mathrm{Fn}}\right)$$

The calculation of the hole density follows by analogy. As the IHB restricts to the sub-band energy levels the integration over the perturbation energy is independent from the momentum space integration. The Gauss–Hermite quadrature is used for numerical integration.

The IHB of the spontaneous emission according to Equation (2) depends on both the conduction and valence band perturbation. However, with reference to the compound disorder it is safe to assume that both perturbations are correlated. The spontaneous emission rate is therefore subject to the common broadening energy $\sigma_{\mathrm{cv}}=\sigma_{c}+\sigma_{v}$. In the Fermi functions the energy perturbation distributes according to the relative contribution of the conduction and valence sub-band. The spontaneous emission rate in presence of the IHB is given by

(7) $$\begin{array}{c} r_{\mathrm{sp}}\left(\hbar \omega ,\vec{e}\right)=\frac{q^{2}\omega n}{\pi \epsilon_{0}m_{0}^{2}\hbar^{2}c^{3}}\sum_{c,v}\int \frac{d^{2}k}{{\left(2\pi \right)}^{2}}{\left|\vec{e}\cdot p_{\mathrm{cv}k}\right|}^{2}\int_{-\infty }^{\infty }d\varepsilon \;L_{\mathrm{cv}}\left(\varepsilon \right)L_{\mathrm{hb}}\left(E_{ck}-E_{vk}+\varepsilon -\hbar \omega \right)\\ \cdot f\left(E_{ck}+\varepsilon \frac{\sigma_{c}}{\sigma_{\mathrm{cv}}}-E_{\mathrm{Fn}}\right)(1-f(E_{vk}+\varepsilon \frac{\sigma_{v}}{\sigma_{\mathrm{cv}}}-E_{\mathrm{Fp}})) \end{array}$$

The integral over the perturbation energy can be eliminated if the homogeneous broadening is insignificant. Integration over the photon energy and the polarisation direction yields the radiative recombination rate

(8) $$\begin{array}{c} R_{\mathrm{sp}}=\frac{q^{2}n}{3\pi \epsilon_{0}m_{0}^{2}\hbar^{2}c^{3}}\sum_{c,v}\int \frac{d^{2}k}{{\left(2\pi \right)}^{2}}{\left|p_{\mathrm{cv}k}\right|}^{2}\int_{-\infty }^{\infty }d\varepsilon \;L_{\mathrm{cv}}\left(\varepsilon \right)\left(E_{ck}-E_{vk}+\varepsilon \right)\\ \;\cdot f(E_{ck}+\varepsilon \frac{\sigma_{c}}{\sigma_{\mathrm{cv}}}-E_{\mathrm{Fn}})(1-f(E_{vk}+\varepsilon \frac{\sigma_{v}}{\sigma_{\mathrm{cv}}}-E_{\mathrm{Fp}})) \end{array}$$

The numerical integration is implemented with the Gauss–Hermite quadrature rule. The quadrature order is moderate allowing efficient integration. We observe an increase of the computation time of approximately 10% due to the IHB model though the calculation of the radiative recombination rate and the Fermi levels occurs within the Newton iteration.

### 4.3. Equivalent Circuit

To support the detailed interpretation of the I/V curve and current contributions in the active region of an an LED an equivalent circuit model has been developed [24]. The equivalent circuit models the ideality factor

(9) $$\eta =\frac{qI}{k_{B}T}\frac{\partial V}{\partial I}$$

and facilitates the analysis of the I/V curve. The concentration of recombination in the QWs and near the contacts as minority recombination gives rise to a lumped model with unipolar currents across the barriers and bipolar currents through the QW and by minority recombination. A schematic of the active region of an SQW LED is illustrated in the inset of Figure 3a. Each circuit element is associated with a dimensionless normalised resistance $\eta_{i}$ related to the differential resistance $r_{i}$:

(10) $$\eta_{i}=\frac{qI_{i}}{k_{B}T}r_{i}=\frac{I_{i}}{k_{B}T}\frac{\partial \Delta E_{F,i}}{\partial I_{i}}$$

Here, $I_{i}$ is the unipolar current through the element or the bipolar current seen through recombination and $\Delta E_{F,i}$ is the QFL difference across the element. The current due to recombination and the distance of the bound QFLs give rise to the normalised QW resistance $\eta_{\mathrm{qw}}$. The barrier resistance $\eta_{b}$ is defined by the distance of the continuum QFLs on either side of the barrier and the unipolar current across the barrier. The scattering circuit element $\eta_{\mathrm{sc}}$ depends on the distance of the bound and continuum QFLs and thus the non-equilibrium distribution. Minority recombination giving rise to carrier leakage is considered by the leakage resistance $\eta_{\mathrm{lk}}$. The calculation of the circuit elements relies on the carrier transport solution. The calculation of ideality by means of the circuit is outlined in [24] and has been found to agree excellently with the ideality factor of the transport simulation.

The equivalent circuit is a valuable tool to investigate the influence of the LED active region on the ideality factor and enables the investigation of current and luminescence re-distribution in an MQW with increasing bias current. Increasing the potential bias the current through the circuit path increases the slower the higher the ideality factor along that path is. Thus, a snapshot of the ideality factor can be directly associated with the distribution of the current amongst the QWs and leakage. Note that an immediate comparison with experimental AlGaN LED results is impeded by the series and contact resistance.

## 5. Conclusions

We have devised a model for the self-consistent modelling of inhomogeneous broadening within a multi-scale and multi-population drift-diffusion framework. The simulation of a simplified single quantum well shows that inhomogeneous broadening due to compound fluctuation in the quantum well affects quantum efficiency as well as the current versus voltage characteristics. The modelling of realistic devices demonstrates that inhomogeneous broadening contributes to the enhanced transversal electric polarised emission observed in deep ultraviolet Aluminium Gallium Nitride light emitting diodes. We are planning an enhancement of the model by including the effect of the Urbach tail.

## Data Availability

Data sharing not applicable.
